# Hemorrhagic Cholecystitis: A Rare Cause of Melena

**DOI:** 10.7759/cureus.16385

**Published:** 2021-07-14

**Authors:** Sreekanthan Gobishangar, John Shelton, Anton A Jenil

**Affiliations:** 1 General Surgery, Faculty of Medicine, University of Jaffna, Jafffna, LKA; 2 General Surgery, Teaching Hospital Jaffna, Jaffna, LKA; 3 Radiology, Base Hospital, Point Pedro, LKA

**Keywords:** hemobilia, melena, hemorrhagic cholecystitis, antiplatelet, interval cholecystectomy

## Abstract

Hemorrhagic cholecystitis -- a rare cause of hemobilia and melena -- is an atypical presentation of calculous cholecystitis, associated with significant morbidity and mortality. A 75-year-old woman with multiple comorbidities, who was undergoing dual antiplatelet therapy, presented with symptoms of acute cholecystitis. Two days later, she developed melena and symptoms of obstructive jaundice. Following radiological evaluation, a diagnosis of hemorrhagic cholecystitis was made. The patient was managed conservatively with IV antibiotics and blood transfusion in the initial period (clopidogrel was withheld); an interval cholecystectomy was performed six weeks later. Hemorrhagic cholecystitis is a rare complication of acute cholecystitis, and its diagnosis is challenging as it mimics various other hepatopancreaticobiliary diseases. Management options include early surgery or conservative management at the initial stage, followed by interval cholecystectomy.

## Introduction

Hemorrhagic cholecystitis is a rare cause of hemobilia and melena [1]. It is associated with significant morbidity and mortality due to delay in its diagnosis. Bleeding into the gallbladder could result from various causes, and the typical presentation of this rare problem only occurs in less than 25% of patients [2]. Therefore, diagnosing hemorrhagic cholecystitis is challenging, as it is often confused with the more common clinical diagnosis of acute cholecystitis [3]. There is no comprehensive study on the management of patients with hemorrhagic cholecystitis, and only case reports and series are available; no specific treatment guidelines exist. Here, we report a case of acute calculous hemorrhagic cholecystitis with melena. The patient had a rare presentation, and thus, her management differed from those reported in most available case reports.

## Case presentation

A 75-year-old woman presented with a two-day history of fever and right upper quadrant abdominal pain. She had a history of diabetes and hypertension and had suffered a myocardial infarction four months prior, for which she was receiving dual antiplatelet therapy (aspirin 75 mg and clopidogrel 75 mg). On clinical examination, the patient was febrile, with a heart rate of 88 beats/min and blood pressure of 140/100 mmHg. There was tenderness on the right side of her abdomen, with a positive Murphy's sign. There were no palpable masses in the abdomen.

One day later, she developed yellowish discoloration of the sclera and melena. Her hemoglobin level was 7.1 g/dL. Other investigations revealed biochemical features of obstructive jaundice (alkaline phosphatase 517 U/L, bilirubin 156.6 µmol/L, direct bilirubin 152.4 µmol/L). Inflammatory marker levels were high (white cell count 16.3 × 109/L, C-reactive protein 61.3 mg/L), and INR was 1.4. She had no history of abdominal trauma or bleeding diathesis.

**Figure 1:**
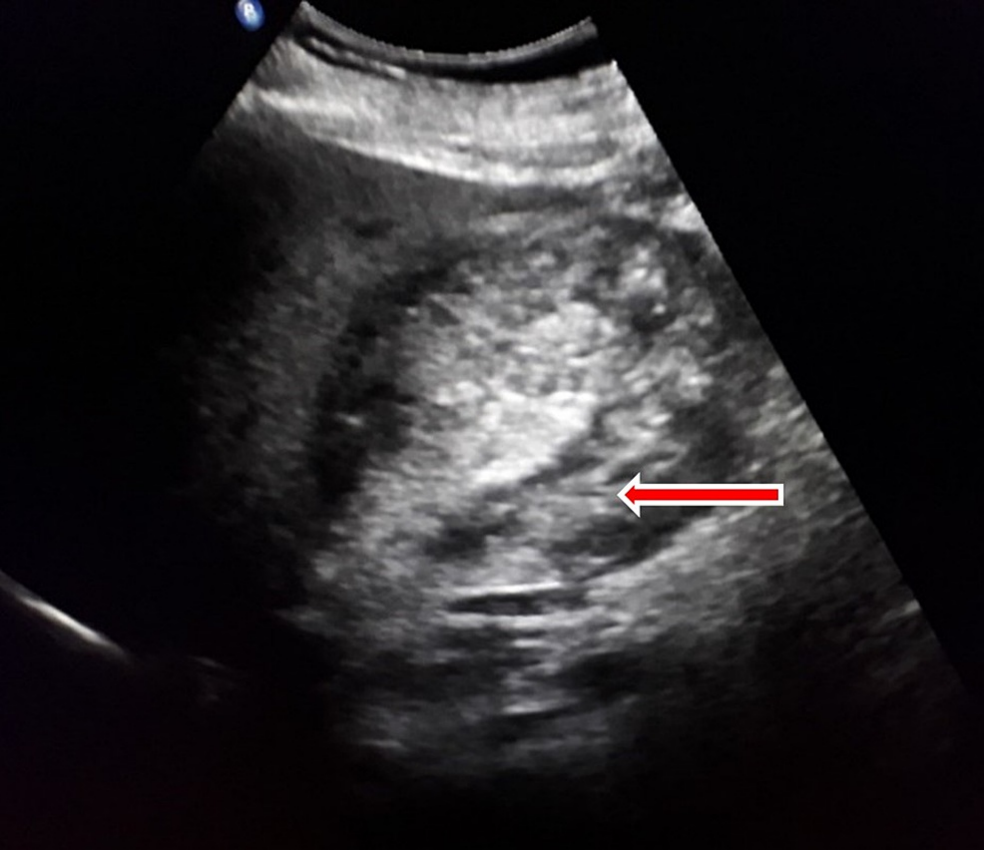
Transabodimal USG of the gallbladder. Mixed echogenic materials and non-acoustic shadowing USG, ultrasonography

**Figure 2:**
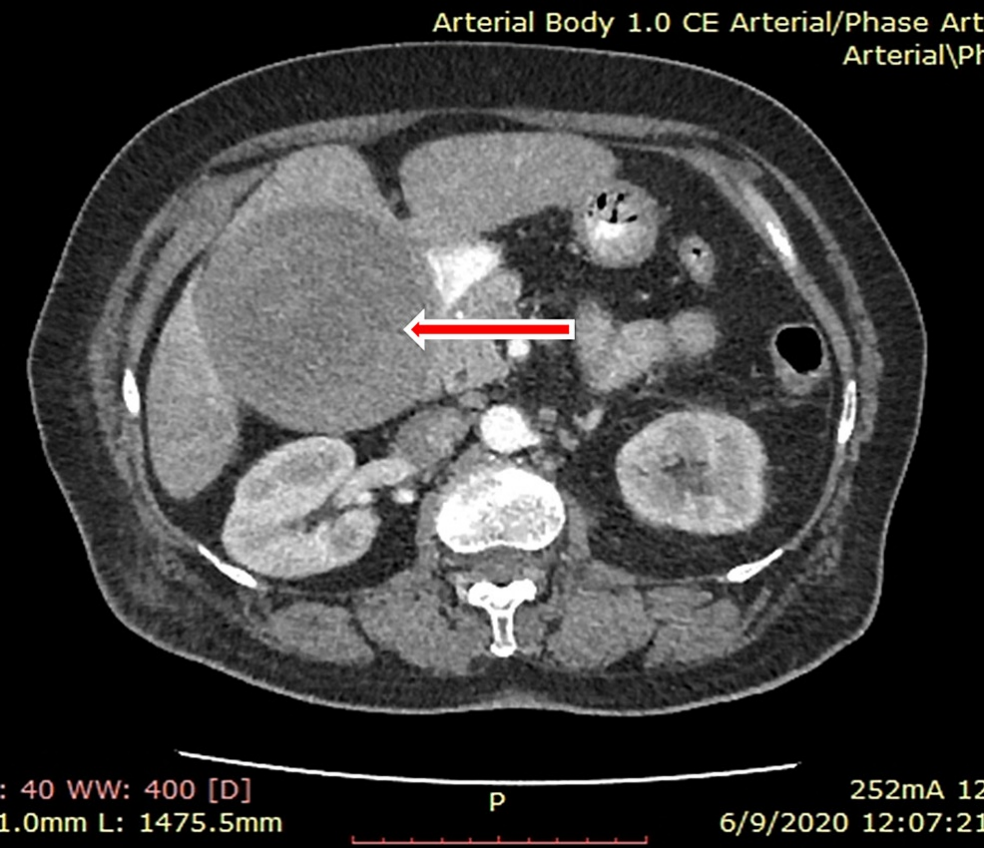
Axial cut of an abdominal CT with contrast. Heterogeneous material with a density almost equal to that of blood filling the entire distended gall bladder, with no significant contrast enhancement

Transabdominal ultrasonography (USG) revealed an avascular material of mixed echogenicity, in a distended gallbladder (Figure 1). Sonographic Murphy’s sign was positive. Non-contrast and IV contrast CT of the abdomen and pelvis showed a heterogeneous material, with a density almost equal to that of blood, filling the entire distended gall bladder, with no significant contrast enhancement in the arterial/venous phases (Figure 2). In addition, CT revealed a small gall stone. There was no biliary dilatation or active arterial bleeding in the biliary system. The patient was diagnosed with hemorrhagic cholecystitis.

 

**Figure 3:**
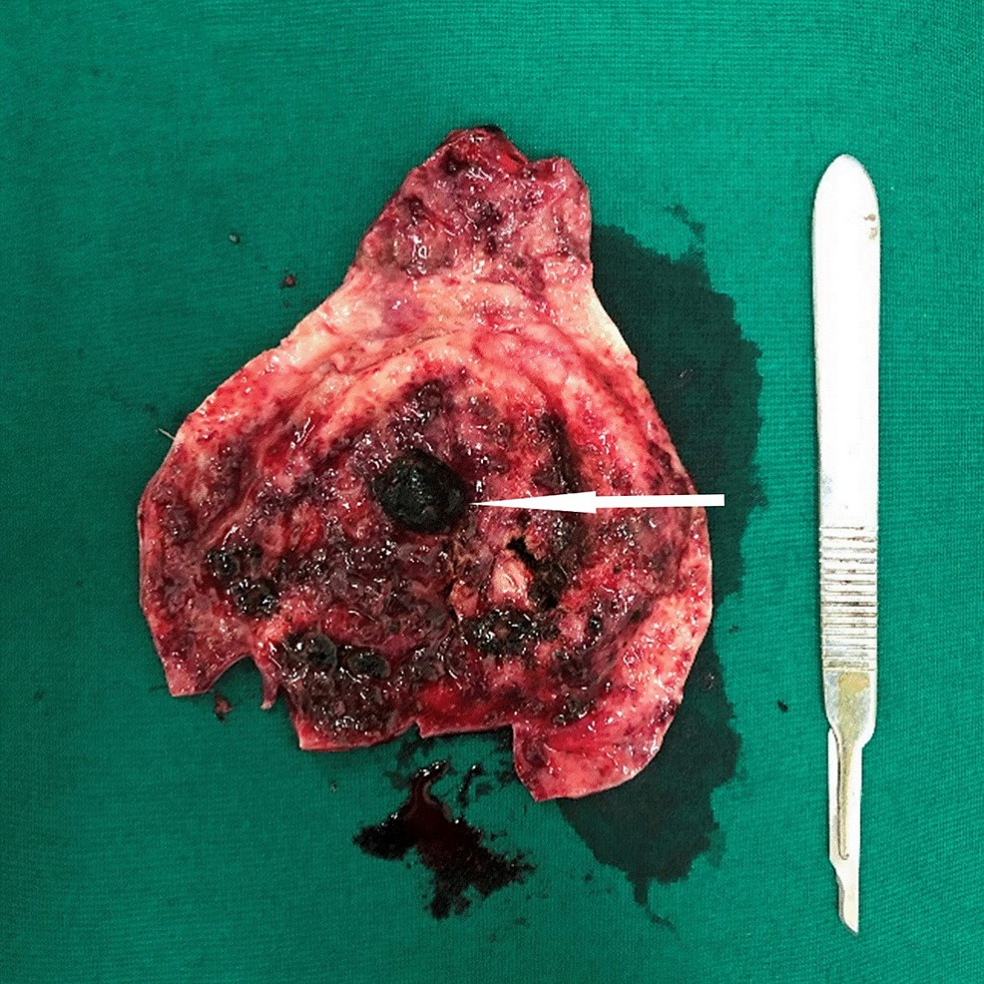
Gross specimen of the gallbladder. Thickened wall shows some residual intraluminal clots with a single calculus indicated by the white arrow

On the day of admission, intravenous cefuroxime and metronidazole were initiated. On day 1, clopidogrel was withheld after the cardiologist’s opinion, and two pints of blood were transfused. On day 3, an esophagogastroduodenoscopy showed no bleeding from the upper gastrointestinal tract and a significant amount of bile in the duodenum. We arranged a multidisciplinary meeting with her physician and cardiologist and decided to continue conservative management unless her condition deteriorated. She responded well to antibiotics, and the melena resolved spontaneously on day 5 of admission. She was discharged after a week of conservative management and underwent laparoscopic cholecystectomy 6 weeks later. The gallbladder wall was thickened and showed some intramural thrombi with a single calculi (Figure 3). The patient had an uneventful postoperative period.

The histology of the gallbladder revealed features of acute on chronic cholecystitis with neither dysplasia nor malignancy. No complications were reported during the follow-up period of six months.

As it is a case report and it is not a clinical study or research, ethical approval was exempted. The patient was managed as it is, and no intervention was done for the publication. Written informed consent was obtained from the patient.

## Discussion

Bleeding into the biliary tract, first described in 1945 by Sandblom, is caused by trauma [4]. Shah and Clegg first published about hemobilia due to hemorrhagic cholecystitis in 1979 [5]. Blood in the gall bladder lumen can be due to trauma, liver biopsy, hepatobiliary instrumentation, biliary tumor, parasite manifestation, aneurysm rupture into the biliary ducts, anticoagulation, or bleeding diathesis (renal failure and cirrhosis) [2, 6-7].

In hemorrhagic cholecystitis, inflammation occurs along the gallbladder wall, leading to mucosal injury and bleeding into the gallbladder lumen [7]. It commonly occurs in patients using antiplatelet (aspirin, cilostazol), nonsteroidal anti-inflammatory, anticoagulant, and steroidal drugs [8-10]. The literature shows that both calculous and acalculous cholecystitis can lead to hemorrhagic cholecystitis [11-12]. Cases of hemorrhagic cholecystitis present in many ways. Sometimes, the gallbladder distends with blood and perforates into the peritoneal cavity, leading to peritonitis, whereas at other times, blood clots may enter the common bile duct and cause obstructive jaundice; moreover, blood may enter the gastrointestinal tract and present as hematemesis or melena (hemobilia) [1].

Hemobilia is a rare presentation of hemorrhagic cholecystitis. Although upper abdominal pain, gastrointestinal tract bleeding, and jaundice (Quinke's triad) are typical characteristics of hemobilia, only 22% of cases have these features [2, 13]. Moreover, patients may have a fever with leukocytosis, and a positive Murphy’s sign [14]. Our patient presented with typical features of hemorrhagic cholecystitis. Blood entered the gastrointestinal tract and the patient had melena.

Abdominal USG is the primary investigation in patients with acute cholecystitis. However, its usefulness in hemorrhagic cholecystitis is questionable. Therefore, diagnosis is made by contrast-enhanced CT. Typical CT findings include thickening of the gallbladder wall, distended gallbladder, and heterogeneous high-attenuation material within its lumen [15-16]. Extravasation of contrast material may be evident in the arterial phase of CT in actively bleeding patients [7]. In addition, endoscopic retrograde cholangiopancreatography (ERCP) is helpful in assessing hemobilia in some patients [17].

According to the literature review of case reports, cholecystectomy is the gold standard treatment for hemorrhagic cholecystitis, and cholecystostomy is suitable in hemodynamically unstable and frail patients [18]. A retrospective analysis by Kim and Kim revealed that ERCP also plays a role in the initial management of biliary obstruction by removing blood clots from the common bile duct. Biliary drainage improves the condition in most cases [17]. However, this condition might be complicated by gallbladder perforation, thereby increasing morbidity and mortality [18]. Our patient was initially managed conservatively by withholding clopidogrel, administering IV antibiotics, and resuscitation with blood and IV fluids.

## Conclusions

In a patient with melena and a typical presentation of hemobilia, hemorrhagic cholecystitis, although rare, should be considered as a differential diagnosis to avoid life-threatening complications. Early diagnosis with clinical and radiological assessment can significantly reduce morbidity and mortality. Although most patients with hemorrhagic cholecystitis can be treated with early cholecystectomy or ERCP, a few hemodynamically stable patients with no active bleeding can be managed conservatively in the initial stage, followed by interval cholecystectomy in six weeks.
